# Volvulus gastrique aigu sur éventration diaphragmatique de l'adulte: à propos d'un cas et revue de la littérature

**DOI:** 10.11604/pamj.2015.20.300.6427

**Published:** 2015-03-30

**Authors:** Mohamadou Lamine Guèye, Alpha Oumar Touré, Ousmane Thiam, Mamadou Seck, Mamadou Cissé, Ousmane Kâ, Madieng Dieng, Cheikh Tidiane Touré

**Affiliations:** 1 Service de Chirurgie Générale, Hôpital Aristide Le Dantec, Dakar, Sénégal

**Keywords:** Volvulus, estomac, occlusion, gastrectomie, diaphragme, Volvulus, stomach, occlusion, gastrectomy, diaphragm

## Abstract

Le volvulus gastrique aigu sur éventration diaphragmatique est une affection rare et une urgence diagnostique et thérapeutique. Sa présentation clinique est peu spécifique et la tomodensitométrie abdominale permet de confirmer le diagnostic. Nous rapportons le cas d'un patient de 22 ans qui présentaitun syndrome occlusif et une voussure épigastrique. A la radiographie de l'Abdomen Sans Préparation, on notait 2 niveaux hydro-aériques sous la coupole diaphragmatique gauche qui était surélevée. Une dévolvulation et une gastrectomie atypique ont été réalisées devant un volvulus gastrique aigu avec nécrose du fundus mis en évidence à la laparotomie. Les suites opératoires ont été simples.

## Introduction

Le volvulus gastrique aigu est une urgence diagnostique et thérapeutique. Il s'agit d'une complication rare de l’éventration diaphragmatique qui en constitue la 2^ème^ étiologie après la hernie hiatale [1]. Il existe plusieurs variétés anatomiques selon l'axe de rotation, le degré de rotation, le siège de l'estomac volvulé et l’étendue de l'atteinte gastrique [2]. Le diagnostic est suspecté à l'examen clinique devant la triade symptomatique de Borchart qui est cependant peu spécifique. La tomodensitométrie abdominale et le transit œso-gastro-duodénal, permettent de confirmer le diagnostic. Le but de ce travail était de discuter les aspects épidémiologiques, diagnostiques et thérapeutiques du volvulus gastrique aigu sur éventration diaphragmatique.

## Patient et observation

**Figure 1:**
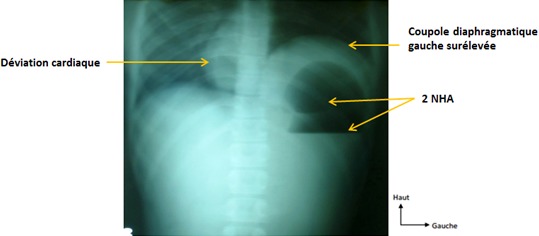
Radiographie de l'abdomen sans préparation

**Figure 2:**
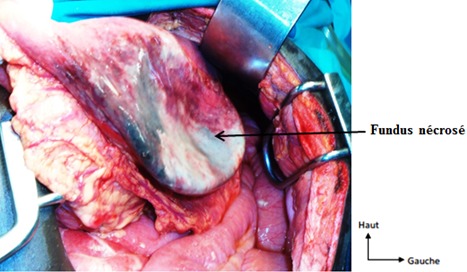
Vue peropératoire de la nécrose fundique

**Figure 3:**
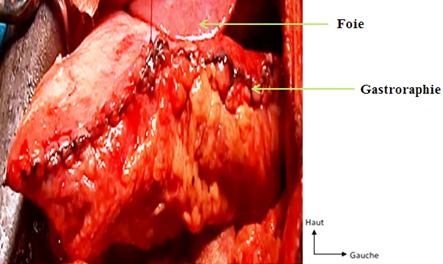
Vue per opératoire de la gastroraphie

Il s'agissait d'un patient de 22 ans, qui a consulté pour des épigastralgies aiguës d'installation brutale, associées à des vomissements liquidiens et à un arrêt du transit digestif. Dans ses antécédents, on retrouvait une notion d’épigastralgies non documentées. A l'examen physique, on notait un assez bon état général, une température à 37,5^°^c, une tachycardie à 140 battements / min, une tension artérielle à 100/70 mmHg, une voussure épigastrique tympanique, un abdomen sensible dans son ensemble avec une défense épigastrique, une ampoule rectale vide et un cul-de-sac de Douglas libre. Le reste de l'examen était sans particularités. La tentative de mise en place d'une sonde nasogastrique était laborieuse. La sonde nasogastrique ramenait 100cc de liquide noirâtre 1 heure après sa mise en place. A la biologie, on notait une hyperleucocytose à prédominance neutrophile à 18200 leucocytes / mm^3^, une hémoconcentration avec une hématocrite à 50,4% et une hémoglobine à 17,4 g/dl, tandis que les plaquettes étaient à 261000/mm^3^. Le taux de prothrombine (TP) était de 61,1%, le temps de Céphaline Activée était de 33,9 secondes pour un témoin de 33 secondes. A la radiographie de l'Abdomen Sans Préparation (ASP) réalisée debout de face, on objectivait 2 niveaux hydro-aériques dans l'hypocondre gauche donnant un aspect de double poche à air gastrique, une surélévation de la coupole diaphragmatique gauche, une grisaille diffuse, une absence de gaz digestif dans le reste de l'abdomen et une déviation de la silhouette cardiaque à droite (Figure 1). Devant l'apparition de signes d'irritation péritonéale, une laparotomie exploratrice a été indiquée. Elle a permis de mettre en évidence: un volvulus gastrique organo-axial à un tour de spire dans le sens horaire, avec une nécrose du fundus (Figure 2). Par ailleurs on notait un liquide péritonéal trouble (100cc), une éventration diaphragmatique gauche, une laxité des ligaments gastriques et une muqueuse gastrique œdématiée et recouverte de dépôts noirâtres. Il n'a pas été retrouvé de hernie hiatale ni d'autres anomalies viscérales. Les gestes chirurgicaux réalisés étaient: une détorsion gastrique, une gastrectomie atypique emportant le fundus nécrosé avec une gastroraphie (Figure 3) et une toilette péritonéale. L'examen anatomopathologique de la pièce opératoire mettait en évidence une nécrose ischémique de la paroi gastrique. Les suites opératoires ont été simples. Aucune complication n'a été notée après un recul de 02 ans.

## Discussion

Le volvulus gastrique est une affection rare; de 1985 à 2008, seuls 757 cas auraient été publiés dans le monde [2]. Dans notre institution, il s'agit probablement du 1^er^cas recensé chez un adulte. Les facteurs étiologiques sont nombreux; l’éventration diaphragmatique venant en seconde position après la hernie hiatale. Le volvulus gastrique est le plus souvent retrouvé chez les sujets âgés, avec un pic de fréquence autour de la cinquantaine [2–5]. Des cas d'adultes jeunes chez qui l’étiopathogénie était dominée par les lésions diaphragmatiques traumatiques ont également été rapportés [2, 6, 7]. Chez notre patient, l’âge jeune et la lésion diaphragmatique était retrouvés, toutefois, l'origine traumatique n'a pu être confirmée. Il pourrait s'agir d'une forme congénitale, d'autant plus que les formes de l'enfant décrites, sont le plus souvent secondaires à des anomalies congénitales [2]. Concernant le genre, il ne semble pas y avoir de prédominance selon la plupart des auteurs [2, 4, 8], même si une prédominance féminine est rapportée par certains [3]. Par ailleurs, chez notre patient, il s'agissait d'un volvulus gastrique partiel, organo-axial antérieur. La variété organo-axiale est la plus fréquente, suivie des formes mésentérico-axiales et des formes mixtes [9]. De même, selon Shivanand, le volvulus partiel serait plus fréquent et comporterait le même risque de strangulation que la forme complète [7]. Toutefois, dans les volvulus partiels, la portion antrale est la plus fréquemment intéressée, ce qui n’était pas le cas chez notre patient. La triade symptomatique de Borchart faite de douleurs épigastriques, d'une distension épigastrique associée à des efforts de vomissements non productifs et d'une impossibilité de mettre en place une sonde nasogastrique était incomplète chez notre patient. Même si elle est évocatrice du diagnostic, cette triade ne serait présente que chez 70% des patients. Le diagnostic clinique de volvulus gastrique sur éventration diaphragmatique peut être suspecté devant des antécédents de dyspnée, même si cela reste anecdotique [10, 11]. En effet, l’éventration diaphragmatique unilatérale est souvent asymptomatique [12, 13]. Son diagnostic est le plus souvent fortuit lors de la réalisation d'une radiographie thoracique, d'un transit œso-gastro-duodénal (TOGD) ou d'une tomodensitométrie abdominale pour un autre motif comme cela a été le cas chez notre patient [12].

En outre, nous n'avons pas retrouvé d'antécédents de dyspnée ni de traumatisme chez notre patient. Il s'agissait sans doute d'une éventration diaphragmatique congénitale. Dans notre observation, seule la radiographie de l'Abdomen Sans Préparation faite debout de face, était réalisée. Elle a permis de suspecter le diagnostic devant une surélévation de la coupole diaphragmatique gauche associée à la présence de 2 niveaux hydro-aériques sous la même coupole donnant l'aspect de « double poche à air gastrique » retrouvé par certains auteurs [10]. La tomodensitométrie abdominale (TDM) voire le TOGD, en l'absence de contre-indications, auraient pu faire poser un diagnostic de certitude avant l'intervention chirurgicale. Par ailleurs, la TDM aurait permis en sus, de déterminer le facteur étiologique, la variété anatomopathologique ainsi que les signes de gravité. Le traitement du volvulus gastrique est chirurgical. Il associe une réduction du volvulus, une prise en charge des complications ainsi que la cure de l’étiologie afin de prévenir la récidive [2]. La laparotomie est la voie la plus utilisée, elle permet un large accès à la cavité abdominale [2]. Chez notre patient, nous avons réalisé une dévolvulation et une gastrectomie atypique par laparotomie. Une gastropexie n'a pas été réalisée et la cure de l’éventration diaphragmatique n'a pas été faite dans le même temps opératoire. Elle est prévue à distance de l’épisode aigu afin de ne pas majorer la morbidité. Cette cure consiste en une phrénoplicature ou une phrénoplastie prothétique. Tout comme certains auteurs, nous n'avons pas réalisé de gastropexie [3, 2, 14]. **Bedioui**, dans une série de 8 cas de volvulus n'avait pas réalisé de gastropexie et ne notait pas de récidive avec un recul de 36 mois [2]. En effet, celle-ci serait facultative en cas d'abord par laparotomie, car les adhérences post-opératoires aboutissent le plus souvent à une gastropexie naturelle [2]. L'abord cœlioscopique dans la cure du volvulus gastrique aigu expérimenté la première fois en 1993 par **Koger**, présenterait des avantages même si les taux de conversion avoisinent les 25% [2, 11, 15]. Dans la série de **Teague** qui regroupait 29 patients, 13 patients ont été opérés par laparotomie, 13 autres par laparoscopie et 3 patients ont bénéficié d'un traitement conservateur [16]. **Teague**, rapportait alors une supériorité de la laparoscopie sur la laparotomie en termes de morbidité et de durée d'hospitalisation [16]. Toutefois, en l'absence d’études prospectives randomisées, cette supériorité n'a pu être démontrée [16]. Enfin, le traitement endoscopique est indiqué chez les malades non opérables. Il consiste en une dévolvulation endoscopique et une gastrostomie percutanée [1]en l'absence de complications locales.

## Conclusion

Le volvulus gastrique aigu est une affection rare et souvent méconnue. Ses étiologies sont multiples et sont dominées par la hernie hiatale et l’éventration diaphragmatique. La tomodensitométrie permet de poser le diagnostic, de trouver l’étiologie et la variété anatomique et de rechercher les signes de gravité. Le traitement est chirurgical et consiste en une dévolvulation, une prise en charge des complications et une cure de l’étiologie afin de prévenir la récidive.
